# *Paracoccus denitrificans* possesses two BioR homologs having a role in regulation of biotin metabolism

**DOI:** 10.1002/mbo3.270

**Published:** 2015-06-02

**Authors:** Youjun Feng, Ritesh Kumar, Dmitry A Ravcheev, Huimin Zhang

**Affiliations:** 1 Department of Medical Microbiology & Parasitology, Zhejiang University School of MedicineHangzhou, Zhejiang, 310058, China; 2 Institute of Biosciences and Technology, Texas A&M Health Science CenterHouston, Texas, 77030; 3 Luxembourg Centre for Systems Biomedicine, University of Luxembourg2, avenue de l’Université, L-4365, Esch-sur-Alzette, Luxembourg

**Keywords:** BioR, biotin, *Paracoccus denitrificans*

## Abstract

Recently, we determined that BioR, the GntR family of transcription factor, acts as a repressor for biotin metabolism exclusively distributed in certain species of *α*-proteobacteria, including the zoonotic agent *Brucella melitensis* and the plant pathogen *Agrobacterium tumefaciens*. However, the scenario is unusual in *Paracoccus denitrificans*, another closely related member of the same phylum *α*-proteobacteria featuring with denitrification. Not only does it encode two BioR homologs Pden_1431 and Pden_2922 (designated as BioR1 and BioR2, respectively), but also has six predictive BioR-recognizable sites (the two *bioR* homolog each has one site, whereas the two *bio* operons (*bioBFDAGC* and *bioYB*) each contains two tandem BioR boxes). It raised the possibility that unexpected complexity is present in BioR-mediated biotin regulation. Here we report that this is the case. The identity of the purified BioR proteins (BioR1 and BioR2) was confirmed with LC-QToF-MS. Phylogenetic analyses combined with GC percentage raised a possibility that the *bioR*2 gene might be acquired by horizontal gene transfer. Gel shift assays revealed that the predicted BioR-binding sites are functional for the two BioR homologs, in much similarity to the scenario seen with the BioR site of *A. tumefaciens bioBFDAZ*. Using the *A. tumefaciens* reporter system carrying a plasmid-borne LacZ fusion, we revealed that the two homologs of *P. denitrificans* BioR are functional repressors for biotin metabolism. As anticipated, not only does the addition of exogenous biotin stimulate efficiently the expression of *bioYB* operon encoding biotin transport/uptake system BioY, but also inhibits the transcription of the *bioBFDA*GC operon resembling the *de novo* biotin synthetic pathway. EMSA-based screening failed to demonstrate that the biotin-related metabolite is involved in BioR-DNA interplay, which is consistent with our former observation with *Brucella* BioR. Our finding defined a complex regulatory network for biotin metabolism in *P. denitrificans* by two BioR proteins.

## Introduction

**Figure 1:**
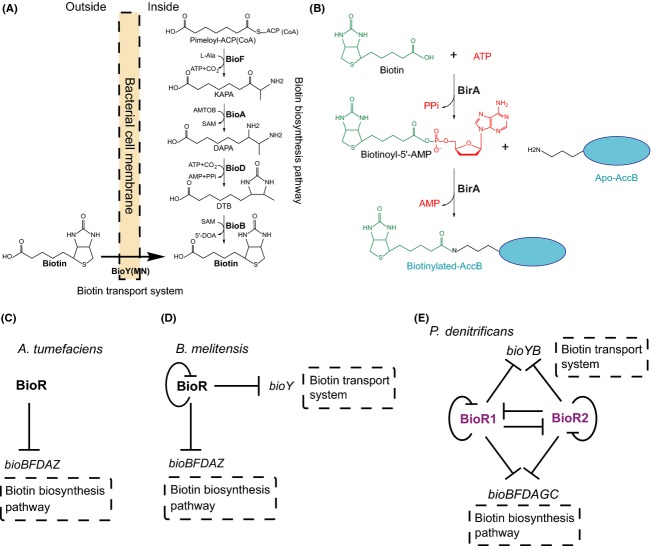
A working model proposed for biotin metabolism and BioR-mediated regulation in *Paracoccus denitrificans*. (A) Schematic diagram for the two bacterial biotin-acquiring strategies (biotin biosynthetic pathway and biotin transport/uptake route). (B) Two half-reactions of BirA-proceeded AccB biotinylation. (C) BioR represses biotin biosynthesis pathway in *Agrobacterium tumefaciens*. (D) Negative autoregulation of BioR and its repression of both biotin biosynthesis pathway and biotin transport system in *Brucella*. (E) Complex regulation network of biotin metabolism by two BioR proteins in *P. denitrificans*. KAPA, 7-keto-8-aminopelargonic acid; DAPA, 7, 8-diaminopelargonic acid; DTB, dethiobiotin; *AMTOB*, S-adenosyl-2-oxo-4-methylthiobutyric acid; SAM, *S*-adenosyl methionine. BioF, 7-keto-8-amino pelargonic acid synthase; BioA, 7,8-diaminopelargonic acid aminotransferase; BioD, dethiobiotin synthase; BioB, biotin synthase; BirA, biotin protein ligase.

Biotin (vitamin H), a sulfur-containing fatty acid derivative, functions as the covalently bound enzyme cofactor that is required by three domains of life (Beckett 2007). The representative biotin-requiring enzyme refers to the AccB subunit (i.e., biotin carboxyl carrier protein, BCCP) of acetyl-CoA carboxylase (ACC), catalyzing the first committed step of fatty acid biosynthesis (Chakravartty and Cronan 2012). To account for such kinds of metabolic requirement for the biotin cofactor, bacteria seemed to have developed two different strategies, one of which is BioY transporter-based scavenging route (Rodionov et al. 2002; Guillen-Navarro et al. 2005; Hebbeln et al. 2007), and the other is de novo synthesis pathway dependent of a full enzyme kit (BioF, BioA, BioD, and BioB) (Fig.1A) (Beckett 2007, 2009). Given the fact that biotin is an energetically expensive molecule in that its de novo biosynthesis requires 20 ATP equivalents, it is reasonable that different organisms have evolved diversified mechanisms to tightly negotiate its production and/or utilization (Streit and Entcheva 2003; Guillen-Navarro et al. 2005; Beckett 2007).

To the best of our knowledge, no less than three types of regulatory factors have been attributed to biotin metabolism (Beckett 2007, 2009; Brune et al. 2012; Feng et al. 2013a,b; Tang et al. 2014). First, the prototypical regulatory mechanism for bacterial biotin synthesis is derived from extensive studies with *Escherichia coli* (Beckett 2007; Chakravartty and Cronan 2012), in which the central player is the bi-functional BirA protein. The *E. coli birA* protein product is unusual, in that it not only functions as a repressor for biotin synthesis route (Barker and Campbell 1981b; Brown et al. 2004; Beckett 2007, 2009), but also acts as the enzymatic activity of biotin protein ligase (BPL) (Fig.1B) (Barker and Campbell 1981a; Cronan 1989; Brown et al. 2004). Given the fact whether the BPL enzyme has the *N*-terminal DNA-binding domain or not, two groups have been categorized (Rodionov et al. 2002). Unlike Group II BPL retaining DNA-binding activity (generally referred to BirA), Group I BPL acts solely as biotin attachment enzymes due to the lacking of the *N*-terminal winged helix-turn-helix DNA-binding motif (Chapman-Smith and Cronan 1999; Henke and Cronan 2014). As the paradigm group II BPL, the *E. coli* BirA thus has the ability to physiologically sense the intracellular levels of both biotin and unbiotinylated biotin accepting protein BCCP (Cronan 1989; Beckett 2005, 2007). Moreover, the regulatory role of *E. coli* BirA depends on the presence of ligand biotinoyl-5′-AMP (biotinyl-adenylate), the product of the first ligase half reaction that is the intermediate of the BirA-catalyzed ligation (Fig.1B) (Ke et al. 2012). Unlike the scenarios seen in *E. coli* carrying the bi-functional BirA regulatory protein, some organisms (e.g., *α*-proteobacteria) only encode Group I BPL with sole ligase activity, suggesting that an alternative transcription factor might exist to compensate the loss of regulatory function for the mono-functional BPL enzyme (Rodionov et al. 2002). This hypothesis was furthered by Rodionov and Gelfand (2006), using the approach of computational prediction. In 2013, we provided integrative experimental evidence that BioR, the GntR family of transcription factor, represses expression of *bio* operon relevant to biotin metabolism in both the plant pathogen *Agrobacterium tumefaciens* (Feng et al. 2013b) (Fig.1C) and the zoonotic agent *Brucella melitensis* (Feng et al. 2013a) (Fig.1D). Relative to the paradigm BirA mechanism that is a single protein model, our findings suggested a new biotin sensing machinery: the two-protein paradigm of BirA and BioR. Very recently, we and others established the second two-protein paradigm for bacterial biotin sensing, in which a new TetR-type transcription factor, referred to BioQ, is recruited in *Mycobacterium smegmatis* (Tang et al. 2014) and *Corynebacterium glutamicum* (Brune et al. 2012). Surprisingly, no direct evidence was found in supporting that DNA binding of BioR (and/or BioQ) requires the participation of biotin metabolites (Feng et al. 2013a,b; Tang et al. 2014), which is far different from scenarios seen with BirA proteins of *E. coli* (Brown et al. 2004; Chakravartty and Cronan 2012) and *Bacillus* (Henke and Cronan 2014).

**Figure 2:**
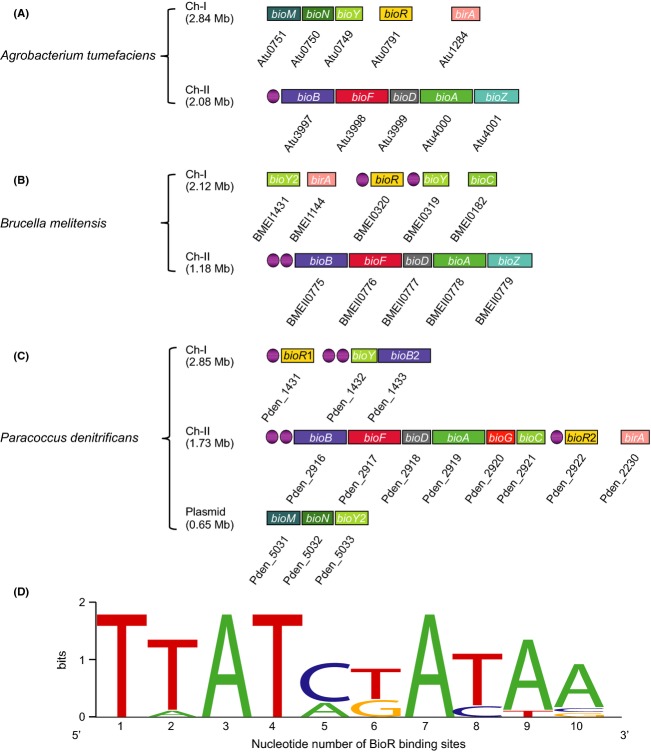
Genetic loci of *bio* operons and BioR signals. (A) Genomic organization of *bio* operon in plant pathogen *Agrobacterium tumefaciens*. (B) Genomic organization of *bio* operon in zoonotic pathogen *Brucella melitensis*. (C) Genomic organization of *bio* operon in *Paracoccus denitrificans*. (D) Sequence logo for the BioR-binidng sites. The sequence logo is generated using WebLogo (http://weblogo.berkeley.edu/logo.cgi).

*Paracoccus* is taxonomically referred to a genus of the Rhodobacteraceae, and comprises a diversified set of species, one of which is *Paracoccus denitrificans* (http://en.wikipedia.org/wiki/Paracoccus) (Ludwig et al. 1993; Rainey et al. 1999). As a nonmotile coccoid soil organism from *α*-subdivision of the phylum proteobacteria, *P. denitrificans* is well known in its unusual ability of denitrification (reducing nitrate to dinitrogen), and growth under the condition of hyper gravity (Baker et al. 1998). The announcement of genomic sequences for *P. denitrificans* such as strain PD1222 (http://genome.jgi-psf.org/parde/parde.home.html) (Siddavattam et al. 2011) greatly facilitated the development of being a model organism for extensive investigations of molecular mechanism (endosymbiotic theory) implicated into denitrifications and possible ancestors for the eukaryotic mitochondrion (http://en.wikipedia.org/wiki/Paracoccus_denitrificans) (John and Whatley 1975). In views of genomic contents, we noted that *P. denitrificans* is unusual in that the gene duplication and/or redundancy (especially two BioR orthologs) is prevalent in the context of biotin metabolism, unlike the scenarios observed with its close relatives *A. tumefaciens* and *B. melitensis* (Fig.2). Also, totally six putative BioR-recognizable palindromes were predicted (http://regprecise.lbl.gov/RegPrecise/regulon.jsp?regulon_id=53141) (Rodionov and Gelfand 2006; Feng et al. 2013a; Novichkov et al. 2013), implying unexpected complexity in BioR-mediated regulation of biotin metabolism in *P. denitrificans*. The question we raised is why *P. denitrificans* evolves such kind of complicated network for biotin metabolism and regulation. Is there any physiological/ecological requirement for this regulatory system in adaptation to its growing/inhabiting niches?

In this paper, we are attempting to address the above questions. We report that (1) extraordinary copies of biotin metabolism-related genes in *P. denitrificans* are acquired through possible events of horizontal gene transfer (HGT); (2) two BioR homologs are functional in biotin regulation/sensing; (3) unprecedent complexity is present in the BioR-mediated regulatory network for biotin metabolism (Fig.1E).

## Experimental Procedures

### Bacterial strains and growth conditions

In addition to PD1222, the wild type of *P. denitrificans*, all of the bacterial strains used here were *E. coli* K-12 derivatives (Table1). The media are separately LB medium (10 g of tryptone, 5 g of yeast extract and 10 g of NaCl per liter), and rich broth (RBO medium; 10 g of tryptone, 1 g of yeast extract, and 5 g of NaCl per liter). Antibiotics were supplemented as follows (in mg/L): sodium ampicillin, 100; kanamycin sulfate, 50; tetracycline HCl, 15; and chloramphenicol, 20.

**Table 1:** Strains and plasmids in this study

Strains or plasmids	Relevant characteristics	References or origins
Strains		
Topo10	A cloning *Escherichia coli* host (F^−^, Δ*lac*X74)	Invitrogen
BL21(DE3)	An expression *E. coli* host	Lab stock
FYJ179	*Agrobacterium tumefaciens* NTL4	Feng et al. (2013b)
FYJ284	NTL4, Δ*bioR*::Km, Δ*bioBFDA*	Feng et al. (2013a,b)
FYJ291	FYJ284 (NTL4, Δ*bioR*::Km, Δ*bioBFDA*) carrying pRG-*PbioB*at	Feng et al. (2013a,b)
PD1222	The wild-type strain of *Paracoccus denitrificans*	ATCC
FYJ347	Topo carrying pET28-*bioR*pd1	This work
FYJ350	Topo carrying pET28-*bioR*pd2	This work
FYJ351	BL21 (DE3) carrying pET28-*bioR*pd1	This work
FYJ354	BL21 (DE3) carrying pET28-*bioR*pd2	This work
FYJ376	FYJ291 carrying pSRKGm-*bioR*pd1	This work
FYJ377	FYJ291 carrying pSRKGm-*bioR*pd2	This work
Plasmids		
pET28(a)	Commercial T7-driven expression vector, Km^R^	Novagen
pET28- *bioR*pd1	pET28(a) carrying *P. denitrificans bioR*pd1 gene, Km^R^	This work
pET28- *bioR*pd2	pET28(a) carrying *P. denitrificans bioR*pd2 gene, Km^R^	This work
pSRKGm	Broad host range expression vector with the tightly regulated promoter	Feng et al. (2013b)
pSRK-*bioR*pd1	pSRKGm encoding *P. denitrificans bioR*pd1 gene, Gm^R^	This work
pSRK-*bioR*pd2	pSRKGm encoding *P. denitrificans bioR*pd2 gene, Gm^R^	This work
pRG970	Low copy transcriptional promoter-less *lacZ/Gus* bi-directional fusion vector, Spc^R^	Van den Eede et al. (1992); van Dillewijn et al. (2001)
pRG-*PbioB*at	pRG970 encoding the *A. tumefaciens bioBFDAZ* promoter region	Feng et al. (2013a,b)

ATCC, American Type Culture Collection.

*Paracoccus denitrificans* (Table1) was grown in minimal medium containing (per liter) 6.0 g of K_2_HPO_4_, 4.0 g of KH_2_PO_4_, 0.15 g of sodium molybdate, 0.2 g of MgSO_4_·7H_2_O, 0.04 g of CaCl_2_, 0.001 g of MnSO_4_·2H_2_O, and 1.1 g of FeSO_4_·7H2O with 1.6 g of NH_4_Cl as the nitrogen source (Zhao et al. 2013; Kumar et al. 2014). Cultures were grown aerobically at 30°C with or without Biotin (100 mmol/L) in mineral medium supplemented with glucose (20 mmol/L) as the carbon source.

### Plasmids and genetic manipulations

The two *bioR* genes (*pden_1431* and *pden_2922*) of *P. denitrificans* were amplified with PCR and cloned into the expression vector pET28(a), giving the recombinant plasmids pET28-*bioR*pd1 and pET28-*bioR*pd2, respectively (Table1). To prepare the appropriate BioR proteins, the corresponding expression plasmids (pET28-*bioR*pd1 and pET28-*bioR*pd2) were transformed into the strain BL21(DE3) (Feng and Cronan 2009b). To examine the role of *bioR* in vivo, the two genes were inserted into pSRKGm, the broad host range expression vector, generating the chimeric plasmids pSRKG-*bioR*1 and pSRKG-*bioR*2, respectively (Table1). The recipient strain is a reporter strain FYJ291 we recently developed (Feng et al. 2013b), which is the Δ*bioR*::Km mutant of *A. tumefaciens* carrying pRG-*PbioB*at, a plasmid-borne LacZ transcriptional fusion (Table1). All the acquired plasmids were confirmed by both PCR detection and direct DNA sequencing.

### Expression and purification of BioR protein

Both BioR1 and BioR2 of *P. denitriifcans* were overexpressed using prokaryotic expression system with induction of 0.3 mmol/L isopropyl *β*-d-1-thiogalactopyranoside (IPTG) at 30°C for 3 h. The clarified supernatant of bacterial lysates was loaded onto a nickel-ion affinity column (Qiagen, Hilden, Germany). After removal of the contaminant proteins with wash buffer containing 50 mmol/L imidazole, the 6x His-tagged protein of interest was eluted in elution buffer containing 150 mmol/L imidazole. The purified proteins were exchanged into 1X PBS buffer (pH 7.4) containing 10% glycerol, and visualized by 15% SDS-PAGE followed by staining with Coomassie Brilliant Blue R250 (Sigma, St. Louis, MO). Of note, the BioR1 is somewhat a weird protein, in that it easily precipitates during the process of purification, which is almost similar to scenarios seen with FabR proteins (Feng and Cronan 2011).

### Liquid chromatography quadrupole time-of-flight mass spectrometry

The identity of two versions of *P. denitrificans* BioR proteins (BioR1 and BioR2) was verified using A Waters Q-Tof API-US Quad-ToF mass spectrometer connected to a Waters nano Acquity UPLC (Feng and Cronan 2011). As we described before (Feng and Cronan 2011), the protein band of interest was digested with Trypsin (G-Biosciences St. Louis, MO), and the resultant peptides were loaded on a Waters Atlantis C-18 column (0.03 mm particle, 0.075 × 150 mm). The dependently acquired data were further subjected to the ms/ms analyses.

### Electrophoretic mobility shift assays

To test the functions of the predicted BioR-binding sites of *P. denitrifican*, gel shift assays were adopted as we described earlier (Feng and Cronan 2009b, 2010, 2011). In addition to the known probe *bioBFDAZ*_ at six more sets of DNA probes were prepared by annealing two complementary oligonucleotides (Table2). These probes included *bioR*1_pd probe, *bioR*2_pd probe, *bioYB*_pd1 probe, *bioYB*_pd2 probe, *bioBFDAGC*_pd1 probe, and *bioBFDAGC*_pd2 probe, respectively (Table2). In the gel shift experiments, the digoxigenin-labeled DNA probes (∼0.2 pmol) were incubated with the purified BioR protein (note: crude extract used for BioR1) in the binding buffer (Roche, Indianapolis, IN, USA) for 15 min at room temperature. When required, the cold probe (and/or biotin metabolites) was supplemented into the gel shift assays. The DNA–protein mixtures were separated with the native 7% PAGE, and transferred onto the nylon membrane via the direct contact gel transfer. Finally, the chemical-luminescence signals were captured through the exposure of the membrane to ECL films (Amersham, GE Healthcare, Piscataway, NJ, USA).

**Table 2:** Primers used in this study

Primers	Sequences (5′-3′)
*bioR*1pd-F (*Bam*HI)	CG *GGATCC* ATG AAA CAC GCC CCT GAA GAG
*bioR*1pd-R (*Xho*I)	CCG *CTCGAG* TTA TCC GGG AAT CTC GTA AGT C
*bioR*2pd-F (*Bam*HI)	CG *GGATCC* ATG AGC GCA GGT TCC GAA GAA
*bioR*2pd-R (*Sal*I)	CCG *GTCGAC* TTA GCC GTG GAT GGC GAA GG
*Pden1431*rt-F	GGC GAC AAT GCC AGT ACC
*Pden1431*rt-R	AGG ATG ATC CGG TGA AAA TG
*Pden1432*rt-F	GCT ATC TGG CGG GCT ATC T
*Pden1432*rt-R	GAG GCC GAG GGC ATA GAC
*Pden1433*rt-F	AGCCTGCTCAGCATCAAGAC
*Pden1433*rt-R	GGATTGCGAGCAATAGCC
*Pden2916*rt-F	CTACAACCACAATATCGACACCTC
*Pden2916*rt-R	ATCCGGTCCTGGAAGGTC
*Pden2917*rt-F	CCTGGTGGTCCATGATGC
*Pden2917*rt-R	GGCATCGTTATGGGCAAA
*Pden2918*rt-F	GGCACCTGCTCTATTTGCAG
*Pden2918*rt-R	CGACAGCAGCGAATGGTT
*Pden2919*rt-F	GGGGCATGTGGTTCTATCAC
*Pden2919*rt-R	GCGATCTCGTCGAAAATCAG
*Pden2922*rt-F	TTCGGCGCCAGCCACGTCCCGGTGC
*Pden2922*rt-R	GTGCGGCGCGGCATGGCGCAGGG
*Pden16S*rt-F	AGGCCCTAGGGTTGTAAAGC
*Pden16S*rt-R	GGGGCTTCTTCTGCTGGTA
*bioB*_at probe-F	CTC TCT TGA GGA GGC AAA AA**T TAT CTA TAA** TTT GCC ATT TAA CGA CCT GC
*bioB*_at probe-R	GCA GGT CGT TAA ATG GCA AA**T TAT AGA TAA** TTT TTG CCT CCT CAA GAG AG
*bioR*1-probe-F^1^	GGT GCA GCA TGA A**TT ATC TAT AA**T TCA TGA AAC ACG
*bioR*1-probe-R^1^	CGT GTT TCA TGA A**TT ATA GAT AA**T TCA TGC TGC ACC
*bioYB*-probe1-F^1^	GAT TCC CGG ATA A**TT ATC TAT AA**A CCT AAT TGC CAG
*bioYB-*probe1-R^1^	CTG GCA ATT AGG T**TT ATA GAT AA**T TAT CCG GGA ATC
*bioYB*-probe2-F^1^	CAA AGC CTT CGT AA**T TAT AGA TAG** ACT CGA TAC CTA TC
*bioYB-*probe2-R^1^	GAT AGG TAT CGA GT**C TAT CTA TAA** TTA CGA AGG CTT TG
*bioBFDAGC-*probe-F^2^	GGC GCT GAC CGT T**TT ATA GAT AC**T TCC ACA TGA GGC
*bioBFDAGC*-probe-R^2^	GCC TCA TGT GGA A**GT ATC TAT AA**A ACG GTC AGC GCC

The underlined sequences in italics are restriction sites, and the bold letters denote the predicted BioR-binding sites.

1 The genetic locus of genes (*bioR*1 and/or *bioBY*) is localized on Chromosome I.

2 The operon of *bioBFDAGC* is localized on Chromosome II.

### *β*-Galactosidase assays

Bacterial samples stripped out of the MacConkey agar plates were suspended in Z-buffer and subjected to direct measurement of *β*-galactosidase activity (Miller 1992; Feng and Cronan 2009a,b). The data were recorded in triplicate more than three independent assays.

### Real-time quantitative polymerase chain reaction

Cells were grown overnight in minimal media without biotin. This was used as an inoculum to inoculate 10 mL of fresh minimal media. Cells were grown upto 0.5 OD_600_ and pelleted and washed with minimal media. Cells were resuspended in 10 mL minimal media and divided into two 5 mL portions. A quantity of 100 nmol/L biotin was added into one portion. Cells were collected at 1/3 h for RNA isolation.

Quantitative real-time PCR was performed as previously described (Pfaffl 2001). Cells were harvested at different OD_600_, and RNA was extracted using RNeasy protect kit (Qiagen) according to the manufacturer’s recommendations. Total RNA was resuspended in PCR-grade nuclease-free water, and RNA quality and concentration were estimated by optical density measurement, using the Nanodrop 2000 (Thermo Fisher Scientific, San Jose, CA, USA). Each sample of 500 ng total RNA was reverse transcribed, using the First Strand cDNA Synthesis Kit (Fermentas, St. Leon-Rot, Germany). Real-time PCR reactions were carried out on a LightCycler 480 (Roche) using the SYBR Green detection format. Change in the expression was calculated relative to the expression of 16S rRNA. After each PCR run, a melting curve analysis was carried out to control for production of primer dimers and/or nonspecific PCR products. Fold change in mRNA expression during treatment was calculated using the crossing point (Cp) for each sample and the efficiency (Eff) of each transcript, using the formula (Eff_target_ gene)ΔCp/(Eff_housekeeping_ gene)ΔCp. The fold change was estimated relative to *16S*rRNA.

### Bioinformatic analyses

The protein sequences of BioR regulators are derived from *A. tumefaciens*, *B. melitensis*, and *P. denitrificans*. The BioR-binding sites were all sampled from RegPrecise database (http://regprecise.lbl.gov/RegPrecise/regulon.jsp?regulon_id=53141). The multiple alignment of protein (and/or DNA) was performed with the program of ClustalW2 (http://www.ebi.ac.uk/Tools/clustalw2/index.html), and the final output of BLAST photography was given after being processed by the program ESPript 2.2 (http://espript.ibcp.fr/ESPript/cgi-bin/ESPript.cgi). The sequence logo of the BioR-specific sites is generated using WebLogo (http://weblogo.berkeley.edu/logo.cgi). Transcription start sites of the *bio* operons were predicted using the method of Neutral Network Promoter Prediction (http://www.fruitfly.org/seq_tools/promoter.html).

Orthologs of BioB, BioR, and BioY proteins were identified by a procedure based on the analysis of phylogenetic trees for protein domains in MicrobesOnline (Dehal et al. 2010). Multiple protein alignments were done using MUSCLE tool (Edgar 2004a,b). Phylogenetic trees were constructed by the maximum-likelihood method with default parameters implemented in PhyML-3.0 (Guindon et al. 2010) and visualized using Dendroscope (Huson et al. 2007).

## Results and Discussion

### Complexity in biotin metabolism of *P. denitrificans*

The situation of genetic organization in *P. denitrificans* seemed to be unusual in that the gene duplication and/or redundancy is present in the context of biotin metabolism and regulation, which is far different from those of its two close-related cousins *A. tumefaciens* and *B. melitensis* (Fig.2 A–C). In addition to the megaplasmid (∼0.65 Mb), *P. denitrificans* also carries two chromosomes (designated as Ch-I (∼2.85 Mb) and Ch-II (∼1.73 Mb), Fig.2C). The *bio* operons in *P. denitrificans* included *bioYB*2 on Ch-1, *bioBFDAGC* on Ch-II, and *bioMNY*2 encoded by the megaplasmid, respectively (Fig.2C). Unlike the *A. tumefaciens* and *B. melitensis* both of which encode only one BioR repressor (Fig.2A and B), *P. denitrificans* has two BioR orthologs (Pden_1431 for BioR1, and Pden_2922 for BioR2) separately scattered on the two chromosomes (Fig.2C) (Rodionov and Gelfand 2006). Additionally, *P. denitrificans* also has two *bioB* homologs (one is located in the *bioBFDAGC* operon, the other is encoded by the *bioYB*2 operon) and two *bioY* paralogs (one is located in the *bioYB*2 operon, the other is encoded by the *bioMNY*2 operon) (Fig.2C) (Rodionov and Gelfand 2006). In much similarity to the scenario seen with *B*. *melitensis bioR* (Fig.2B) (Feng et al. 2013a), the two *bioR* homologs of *P. denitrificans* each has a putative BioR-specific palindrome in front of their coding sequences (Fig.2C and D), suggesting the possibility of autoregulation. No putative BioR-binding site was detected in the plasmid-borne *bioMNY*2 operon (Fig.2C), which is in much consistency with the scenario with the *A. tumefaciens bioMNY* (Fig.2A) (Feng et al. 2013b). In contrast, the other *bioY*-containing operon *bioYB* seemed likely to be controlled by the BioR regulator, in that it has two tandem BioR-recognizable sites (Fig.2C and D). As anticipated, the *bioBFDAGC* operon, a major gene cluster encoding the full de novo biotin synthesis pathway also has two tandem BioR-binding sites (Fig.2C), which is almost identical to the observation with *B. melitensis bioBFDAZ* (Fig.2B) (Feng et al. 2013a), but little bit different from that of the *A. tumefaciens* counterpart having only one palindrome for the BioR protein (Fig.2A) (Feng et al. 2013b). Of particular note, the *bioZ* gene is replaced with *bioGC* in this case (Fig.2A–C). Given the fact that two BioR homologs and 6 BioR-recognizable sites (representing 4 target genes/operons) coexist, we concluded that the BioR-mediated regulatory network in *P. denitrifican* is of unusual complexity (Fig.1E).

### Tracing origins of *bio* operons/genes of *P. denitrificans*

Since the situation of *bio* operons/genes is pretty unusual in *P. denitrificans*, we are interested in tracing the origins of these genes *esp*. the duplicated cousins. The BLAST analyses revealed that the *bio* operons/genes of *P. denitrificans* can match no less than eight different species, including the plant pathogen *Xylella fastidiosa* and the marine bacteria *Celeribacter indicus* (Table3). Of being noteworthy, the *P. denitrificans bioG* is completely identical to the *X. fastidiosa* counterpart at the level of nucleotide acids (Table3). Systematic comparison of the GC contents showed that (1) *bioR*2 (*pden_2922*) with the GC percentage of 72.52% (but not *bioR*1 (*pden_1431*) with 66.36% of GC percentage) is significantly higher than that of the average GC% of the chromosome (66.7–66.8%); (2) *bioY*1 (*pden_1431*) with the 71.86% of GC percentage (but not *bioY*2 (*pden_5033*) with the GC percentage of 68.92%) is appreciably higher than that of the average GC% of the chromosome/megaplasmid (66.7–67.1%); (3) the group I BPL-encoding gene *birA* (*pden_2230*) exhibits the GC ratio of 72.6%, much higher than that of the Chromosome II (66.8%); (4) most of genes encoding the biotin synthesis pathway consistently showed higher GC% (74.37% for *bioF*, 74.06% for *bioD*, 71.18% for *bioA*, and 75.13% for *bioC*) than that of Chromosome II (66.8%), except that *bioG* presents 54.64%, the lowest GC% amongst the *bio* genes (Table3). Obviously, the above observations might indicate the possibility for HGT in the context of biotin metabolism-related gene clusters/operons. We anticipated that the heterogeneity (heterogeneous origins) somewhat is in part (if not all) why *P. denitrificans* evolves such kind of complicated machinery for biotin metabolism. However, the physiological/ecological advantage of this unusual mechanism requires further explorations.

**Table 3:** GC% analyses of the *Paracoccus denitrificans bio* operon and exploration of their possible origins

	GC%			Origins matched^1^
	Ch-I	Ch-II	Plasmid	
Ch-I	66.7	–	–	–
Ch-II	–	66.8	–	–
Plasmid	–	–	67.1	–
*birA*	–	72.6	–	*Paracoccus aminophilus* (76%)
*bioR*1	66.36	–	–	*Celeribacter indicus* (84%)
*bioR*2	–	72.52	–	*Azorhizobium caulinodans* (78%)
*bioB*1	–	66.77	–	*Rhodobacter capsulatus* (81%)
*bioB*2	68.22	–	–	*Rhodobacter sphaeroides* (90%)
*bioY*1	71.86	–	–	*Paracoccus denitrificans* (100%)
*bioY*2	–	–	68.92	*P. denitrificans* PD1222 plasmid 1 (100%)
*bioM*	–	–	66.54	*P. denitrificans* plasmid 1 (100%)
*bioN*	–	–	70.23	*P. denitrificans* plasmid 1 (100%)
*bioF*	–	74.37	–	*P. denitrificans* (100%)
*bioD*	–	74.06	–	*P. aminophilus* (70.9%)
*bioA*	–	71.18	–	*R. capsulatus* (76%)
*bioG*	–	54.64	–	*Xylella fastidiosa* (100%)
*bioC*	–	75.13	–	*P. denitrificans* (100%)

–, not applicable; Ch, chromosome.

1 The nucleotide identity of the interested gene from *P. denitrificans* relative to its possible origins. The numbers in grey background denote the GC percentage of P. denitrificans Chromosome/plasmid.

**Figure 3:**
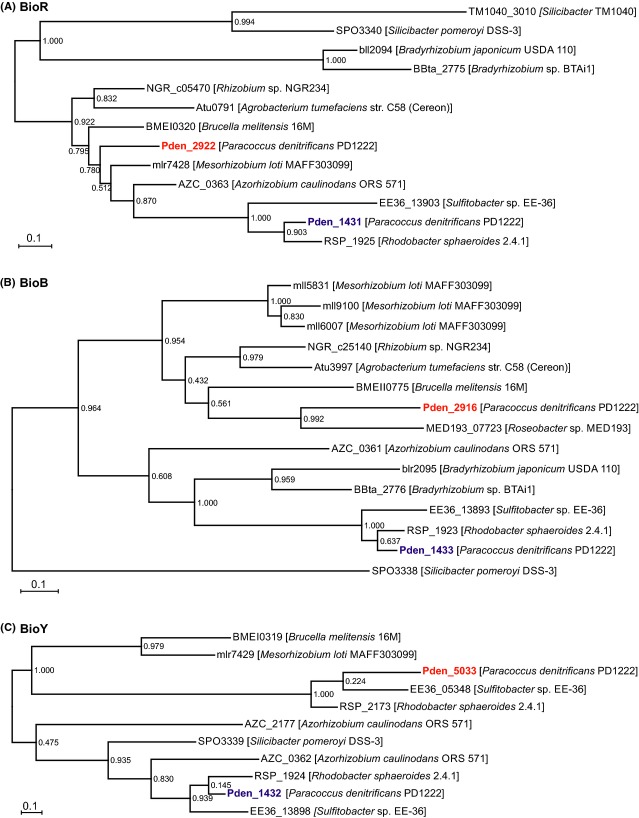
Phylogenetic trees for the biotin synthesis proteins duplicated in *Paracoccus denitrificans* and their orthologs in Rhodobacterales and Rhizobiales genomes. (A) Phylogenetic tree of the BioR homologs. (B) Phylogenetic tree of the BioB proteins. (C) Phylogenetic tree of the BioY transporters.

For better understanding of origin of the duplicated genes, *bioR* (Fig.3A), *bioB* (Fig.3B), and *bioY* (Fig.3C), we analyzed their orthologs in genomes of Rhizobiales and Rhodobacterales. These phylogenetic analyses revealed that at least *bioR1* gene (*pden_1431*) and *bioYB2* operon (*pden_1432-33*) might be products of the horizontal transfer from *Azorhizobium caulinodans* or the related species (Fig.3).

### Characterization of two BioR homologs

**Figure 4:**
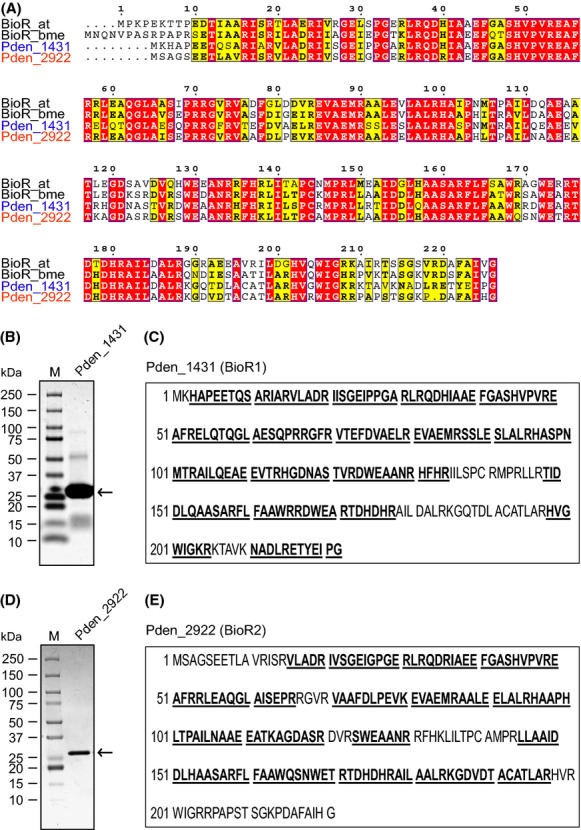
Characterization of the two BioR homologs of *Paracoccus denitrificans*. (A) Multiple sequence alignments of the the two BioR homologs *of P. denitrificans* with the paradigm members The multiple alignment of bacterial BioR homologs was performed using ClustalW2 (http://www.ebi.ac.uk/Tools/clustalw2/index.html), and the final output was expressed after data processing by program ESPript 2.2 (http://espript.ibcp.fr/ESPript/cgi-bin/ESPript.cgi). Identical residues are in white letters with red background, similar residues are in black letters in yellow background, and the varied residues are in gray letters. SDS-PAGE profile (B) and MS-identification (C) of the purified BioR1 (Pden_1431) protein SDS-PAGE profile (D) and MS-identification (E) of the purified BioR2 (Pden_2922) protein.

*Paracoccus denitrifIcans* PD1222 contains two circular chromosomes: Ch-I (Accession no.: NC_008686.1) is 2.85 Mb long, while Ch II (Accession no.: NC_008687.1) is 1.73 Mb in length (http://www.ncbi.nlm.nih.gov/genome/?term=PD1222). Two *bioR* homologs separately are localized on the corresponding chromosome: *bioR*1 (*pden_1431*) on Ch-I encodes a 222 residues of polypeptide, whereas the *bioR*2 (*pden2922*) on Ch-II is a gene encoding a protein of 221 aa long (Figs.2C and 4A). Multiple sequence alignments of the two BioR proteins (BioR1 & BioR2) with the cousins of both *A. tumefaciens* and *B. melitensis* showed that they are appreciably conserved (Fig.4A). Given the very fact that BioR1 and BioR2 both share 76.1% identity and 66.7% similarity, respectively (not shown), we cannot figure out which one is the ancestor of the two duplicated *bioR* genes. Subsequent measurement for the GC contents of the two *bioR* genes ruled out the hypothesis that they are generated during the events of gene duplication in that the difference in GC% (66.36% for *bioR*1, and 72.52% for *bioR*2) raised the possibility that they are acquired by gene horizontal transfer (Table3). Further BLAST analyses indicated that *bioR*1 might be derived from *C. indicus*, whereas *bioR*2 can be traced to *A. caulinodans* (Table3).

As predicted by Rodionov and Gelfand (2006), structural modeling suggested that the two BioR proteins are featuring with a conserved *N*-terminal DNA-binding motif with helix-turn-helix structure (not shown). To examine the putative function, we produced the two versions of recombinant BioR1 (and/or BioR2) protein in the *E. coli* expression system and purified them to homogeneity (Fig.4B and D). As anticipated, the two BioR proteins are weird (not easily tractable), in that most of them precipitates during the process of protein purification in vitro. The similar scenarios notorious in short survival time of protein were ever encountered in the cases of *A. tumefaciens* BioR (Feng et al. 2013b) and the counterpart of *B*. *melitensis* (Feng et al. 2013a). Subsequently, the two protein bands cut from the SDS-PAGE gel was subjected to the liquid chromatography mass spectrometry. The MS results of the resultant tryptic peptides showed that BioR1 (Fig.4B) and BioR2 (Fig.4D) we overexpressed in vitro well matched Pden_1431 with the coverage of 81% (Fig.4C), and Pden_2922 with the covering score of 72% (Fig.4E), respectively. Fortunately, we have luck to recover around 10% of soluble BioR2 protein, whereas we do not have any success to acquire trace amount of BioR1 protein even after a series of trials (that is why we have to fall back on the crude extract containing BioR1 protein for subsequent functional assays).

### Binding of *P. denitrificans* BioR cognate genes

We performed an extensive bioinformatics analyses, using The Neutral Network Program of Promoter Prediction (http://www.fruitfly.org/seq_tools/promoter.html), which roughly illustrated the promoters of *bio* operons/genes (Fig. S1). Totally, six BioR-recognizable sites are assigned to four genes/operons: the two *bioR* each has one site, the two gene clusters (*bioYB*2, and *bioBFDAGC*) each has two discontinuous sites (Fig.2C and D). Prior to this study, we believed that the situation of biotin regulation in *B. melitensis* is quite complicated when compared with that of *A. tumefaciens* (Fig.1C and D). It seemed likely that the scenario is much more complex in the closely related organism *P*. *denitrificans*.

**Figure 5:**
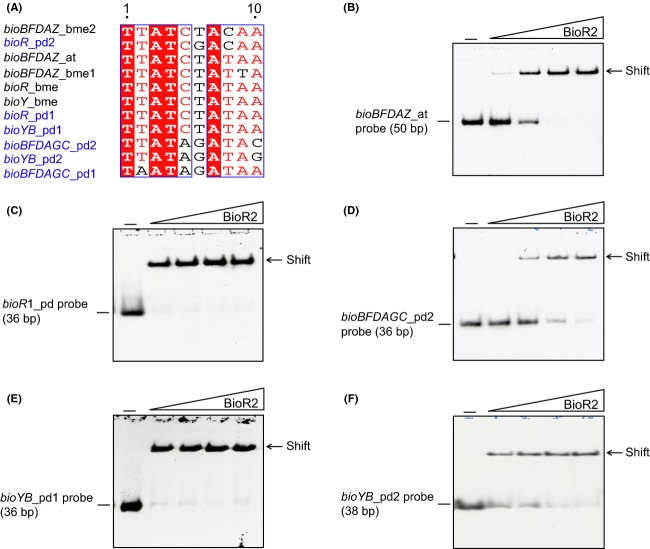
Binding of *Paracoccus denitrificans* BioR2 (Pden_2922) to cognate target genes. (A) Comparative analyses for the BioR-recognizable sites. (B) Binding of *Agrobacterium tumefaciens bioBFDAZ* promoter to the *P. denitrificans* BioR2 (Pden_2922) protein. (C) Binding of *P. denitrificans bioR*1 (*pden_1431*) promoter to the *P. denitrificans* BioR2 (Pden_2922) protein. (D) *P. denitrificans* BioR2 (Pden_2922) protein interacts with the promoter of *P. denitrificans bioBFDAGC* operon Interplay between *P. denitrificans* BioR2 (Pden_2922) protein and the two putative sites of the *bioYB* operon, one of which is *bioYB*1 (E) and other is *bioYB*2 (F). at, *Agrobacterium tumefaciens*; bme, *Brucella melitensis; pd, Paracoccus denitrificans*..

To test the functions of these predicted BioR sites (Fig.5A), electrophoresis mobility shift assay (EMSA) was conducted using the either the purified BioR2 protein (Fig.5B–F) or the crude extract containing BioR1 protein (Fig. S2). Gel shift assays confirmed that BioR2 protein effectively bind the probe of *A. tumefaciens bioBFDAZ* operon in a dose-dependent manner (Fig.5B), which is generally similar to our former observation with BioR proteins of both *A. tumefaciens* and *B. melitensis* (Feng et al. 2013a,b). The appreciable binding of the same *bioBFDAZ_at* probe to BioR1 protein was also confirmed (Fig. S2A). Obviously, it suggested that BioR homologs with a variety of origins are functionally exchangeable. The promoter of *bioR*1 interacted well with the BioR2 protein (Fig.5C) as well as the BioR1 protein (Fig. S2B), and vice versa (not shown). This implied that not only do the two regulators (BioR1 & BioR2) autoregulate themselves, but also they can crosstalk via direct DNA–protein interaction. As expected, the *bioBFDAGC* promoter of *P. denitrificans* exhibited strong binding to the BioR2 (Fig.5D) and BioR1 (Fig. S2C), validating the speculation by Rodionov and Gelfand (2006) that the biotin biosynthetic route is under the control by the BioR regulatory protein. Unlike the scenarios in *A. tumefaciens* (Feng et al. 2013b) and *B. melitensis* (Feng et al. 2013a), the situation in the case of *P. denitrificans* seemed unusual, in that two BioR (BioR1 and BioR2) transcription factors constitute a “double-safety locker” to guarantee the tight regulation exerted on the biotin synthesis pathway. In addition, the promoter region of the *bioYB* operon was found to bind both BioR2 (Fig.5E and F) and BioR1 (Fig. S2D). To the best of our knowledge, it might represent the second example of the BioR-regulated transport/scavenge of biotin in bacteria, in that the first paradigm was attributed to its close relative, the human pathogen *B. melitensis* (Feng et al. 2013a).

**Figure 6:**
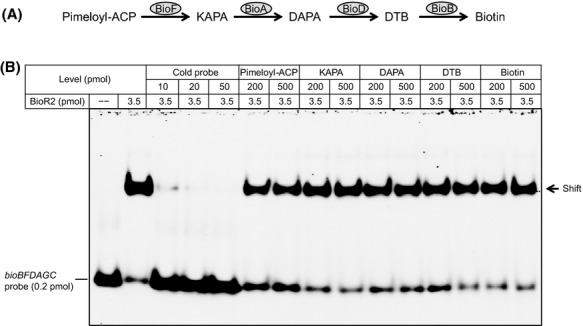
Probing possible roles of biotin-related metabolites in the specific interaction between BioR and cognate DNA. (A) Schematic diagram for the four-step pathway of bacterial biotin biosynthesis. (B) The binding of BioR cognate DIG-labeled DNA can be fully/specifically interfered with the excess of the relevant cold DNA probe, but no apparent roles of biotin metabolites (Pimeloyl-ACP, KAPA, DAPA, and DTB) are seen in such kinds of interaction. The minus sign denotes no addition of BioR2 protein (10–20 pmol). The protein samples were incubated with 0.2 pmol of DIG-labeled *bioBFDAGC* probe in a total volume of 15 *μ*L. When required, the cold *bioBFDAGC* probe is supplemented at different levels (10, 20, and 50 pmol). A representative result from no less than 3 independent gel shift assays (7% native PAGE) is given. KAPA, 7-keto-8-aminopelargonic acid; DAPA, 7, 8-diaminopelargonic acid; DTB, dethiobiotin. BioF, 7-keto-8-amino pelargonic acid synthase; BioA, 7,8-diaminopelargonic acid aminotransferase; BioD, dethiobiotin synthase; BioB, biotin synthase; BirA, biotin protein ligase.

Although the most straightforward model for BioR regulation referred to that BioR binding its cognate operators requires coexistence of either biotin or a biotin derivative such as biotinoyl-5′-adenylate, unfortunately we are still not aware of any direct evidence thus far. To address the long-term unresolved question, potential effectors/ligands for the DNA-BioR interplay, we systematically tested the precursor (pimeloyl-ACP), intermediates (KAPA, DAPA, and DTB), and the final product (biotin) of biotin synthesis pathway (Fig.6A) by employing EMSA approach. In much agreement with the scenarios seen with the BioR proteins of *A. tumefaciens* (Feng et al. 2013b) and *B. melitensis* (Feng et al. 2013a), we failed to visualize that the biotin-related metabolites we tested have obvious roles in interfering with the DNA-binding activity of BioR2 protein even after addition of excess metabolites (such as 500 pmol KAPA, DAPA, DTB, and biotin) (Fig.6B). As anticipated, we also noted that excess of cold DNA probe competitively impaired interplay of the DIG-labeled *bioBFDAGC* probe and BioR2 protein (Fig.6B), demonstrating that binding of BioR2 cognate DNA is a specific physical interaction.

### *In vivo* role of BioR regulatory protein

**Figure 7:**
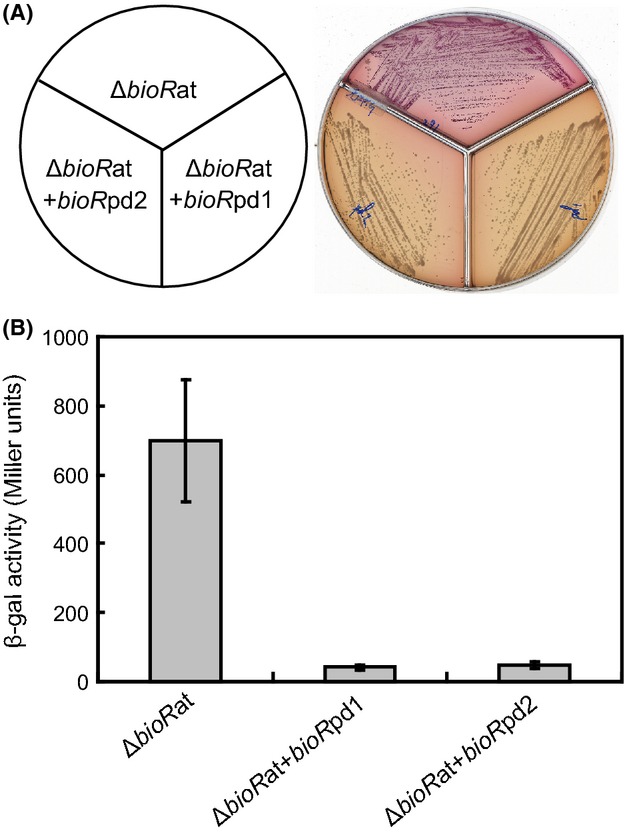
Functional analyses for two *bioR* homologs of the *Paracoccus denitrificans* using the *Agrobacterium tumefaciens* reporter system. (A) Maconkey plates-based assays for the activities of the two *P. denitrificans* BioR homologs (Pden_1431 and Pden_2922). (B) Measurment for *β*-gal activities of the Pden_1431 (and/or Pden_2922) promoter-driven LacZ reporter genes. Three strains used here include FYJ291 (NTL4, Δ*bioR*::Km, carrying pRG-*PbioB*at); FYJ376 (FYJ291 carrying pSRKGm-*bioR*pd1) and FYJ377 (FYJ291 carrying pSRKGm-*bioR*pd2), respectively.

Very recently, we developed a reporter strain FYJ291, a Δ*bioR* mutant of *A. tumefaciens* engineered to carry the low-copy plasmid-borne P*bioB*at*-lacZ* transcriptional fusion (Table1) (Feng et al. 2013a,b). This reporter strain has been confirmed to work well in identifying the functional *bioR* ortholog in vivo (Feng et al. 2013a). In principle, growth of the reporter strain FYJ291 on a MacConkey agar plate with 0.2% lactose as a sole carbon source can give purple colonies, implying the robust *β*-gal activity by P*bioB*at*-lacZ* fusion is present upon removal of BioR repressor (Fig.7A). The introduction of both *bioR*1 and *bioR*2 into this reporter strain caused the formation of yellow colonies, suggesting that the expression of either BioR1 or BioR2 can downregulate *β*-gal activity of the P*bioB*at*-lacZ* fusion (Fig.7A). Indeed, such dramatic color alterations generally agreed with our former observations with BioR of *A. tumefaceins* (Feng et al. 2013b) and *B. melitensis* (Feng et al. 2013a) in this bioassay. Analyses for LacZ activities further showed that expressions of both *bioR*1 and *bioR*2 of *P. denitrificans* give a six to eightfold decrement of the *bioB_*at transcription level in comparison with that of FYJ291 indicator strain (Fig.7B). Therefore, both *bioR*1 and *bioR*2 of *P. denitrificans* encode a functional BioR ortholog having the in vivo role in modulating biotin metabolism.

### Biotin sensing of *P. denitrificans*

**Figure 8:**
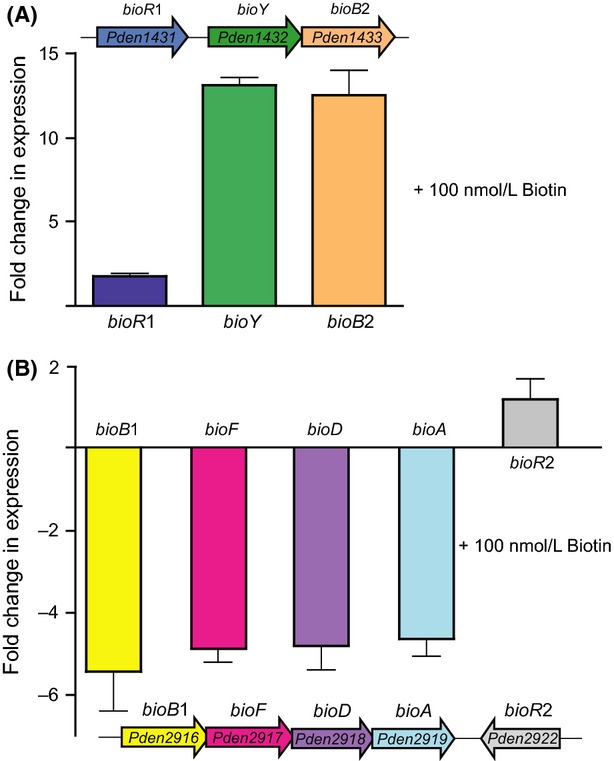
Alteration of *bio*expression by addition of biotin. (A) Induction of the *bioY*-containing operon *bioYB*2 by addition of exogenous biotin. (B) Repression of the biotin biosynthesis operon *bioBFDA* in the presence of excess of biotin The inside cartoon diagram illustrates the genetic organization of *bio* operons. 100 nmol/L biotin was added into the *Paracoccus denitrificans* grown in minimal medium, and real-time quantitative PCR is used to measure the relative expression of biotin-related genes.

We carried out qPCR assays to investigate the response of *P. denitrificans* to biotin by addressing the accumulated transcript level of the representative target genes that correspond to the two biotin-acquiring systems (*bioY* is for biotin uptake system, and *bioB*, *bioF*, *bioD*, and *bioA* are specific for biotin synthesis pathway, Fig.8). First, we observed that an addition of exogenous biotin (100 nmol/L) to cultures of the wild-type strain PD1222 gave more than 10-fold increment to transcription of the *bioYB*2 operon, but did not alter significantly the expression of *bioR*1 (Fig.8A). Somewhat, it seemed usual in that the increasing expression of BioB, an enzyme catalyzing the last committed step of biotin synthesis is energetically wasteful on the condition with the supply of exogenous biotin. Someone might conclude that it is not physiologically correct that the *bioB*2 is co-transcribed with *bioY* forming an operon of *bioYB*2. In contrast, we favored to believe it is possible. The reasons being in the following two points: (1) Although all the intermediates can enter *E. coli* at various efficiencies, the biotin transporter BioY might be helpful for uptake of DTB, a precursor for biotin (also the substrate of BioB biotin synthase); (2) when the DTB is available, expression of functional BioB is physiologically required to make biotin from DTB. Given the fact that no literature documented the above speculation thus far, it would be of much interest to test it. This hypothesis might be checked by seeing if DTB competes with biotin in a *bioB* strain. The criteria for this assay is described as follows: If DTB uses the same transporter, then the minimal amount of biotin will not be enough (this idea is mainly from personal communication with Prof. John Cronan, University of Illinois at Urbana-Champaign, Urbana, IL).

Consequently, the supply of exogenous biotin (100 nmol/L) to bacterial cultures resulted in around fivefold decrement to expression of *bioBFDAGC* (note: the former four genes of this operon *bioB*, *bioF*, *bioD*, and *bioA* were checked), but no obvious change in *bioR*2 transcription. It demonstrated that the presence of biotin can effectively shut down the biotin synthesis pathway (Fig.8B). Together, the altered expression profile observed with *P. denitrificans* in responding to biotin is expected to be physiologically relevant.

## Conclusions

The data shown here represented a first paradigm that the crosstalk between two functional BioR regulators is involved in modulating bacterial biotin metabolism. The BioR-mediated regulatory network for biotin metabolism is unprecedent, complicated/complex in that no less than four aspects are involved (Fig.1). Briefly, (1) The two BioR (BioR1 and BioR2) are autoregulators; (2) BioR1 and BioR2 can crossregulate each other; (3) BioR1 (and/or BioR2) can repress the *bioY* biotin transporter-containing *bioYB*2 operon; (4) BioR1 (and/or BioR2) negatively regulates the *bioBFDAGC* operon encoding the full biotin synthesis pathway (Fig.1). Given the fact that *birA* of *P. denitrificans* only encode a Group I BPL lacking the DNA-binding motif, it is reasonable that BioR, a novel GntR-like transcription factor, is evolved to compensate for the loss of regulatory function of BirA, a monofunctional BPL. As we earlier proposed (Feng et al. 2013a,b), we still favored a two-protein model of BirA and BioR, which represents an alternative mechanism for bacterial biotin sensing. The dramatic GC% difference of the two *bioR* homologs argues greatly the prediction that the event of *bioR* duplication exists in *P. denitrificans* (Table3). The fact that the number of BioR sites in *P. denitrificans* is most also determines in part the complexity in regulation of biotin metabolism by BioR.

Somewhat it seemed unexpected that the addition of exogenous biotin exerted an opposite effect on biotin biosynthesis operon *bioBFDAGC* and biotin transporter-containing operon *bioYB* in that it is quite different from the perspective of other biotin regulons in different bacterial species. For instance, BirA is a repressor of both biotin biosynthesis and transport genes in *Bacillus sphaericus* and other *Firmicutes* (Bower et al. 1995, 1996). Recently, BirA was found to act as a repressor of the novel biotin transporter *yigM* in *E.coli* (http://epub.uni-regensburg.de/15822/). BioQ in *Actinobacteria* also acts a repressor of both biotin biosynthesis and transporter operon (Brune et al. 2012; Tang et al. 2014). Moreover, even in yeasts, it has been shown that in addition to the transport genes, low biotin concentrations result in increased levels of transcription of the biosynthetic genes as well as the gene that encodes the BPL (Pirner and Stolz 2006; Beckett 2007).

We still have no success in identifying the possible ligands for BioR binding to cognate promoters (Fig.6). Crystallization of BioR alone and bound DNA might be a direct way to visualize if the ligand molecule is present or not. However, this approach seemed not easy in that the BioR protein is weird/hard tractable (precipitate at high level). In fact, we are frustrated by the in vitro performance of BioR proteins from three different organisms (*A. tumefaciens*, *B. melitensis*, and *P. denitrificans*) to some extent. The other possibility might be some unknown signaling pathway is linked to BioR-mediated regulation mechanism. While no evidence supports the above hypothesis right now. In summary, the existence of two BioR homologs in *P. denitrificans* defines a complex regulatory network, augmenting the diversity in the context of bacterial biotin metabolism.

## Supporting Information

**Figure S1.** Molecular dissection for promoters of the *bio* operons from *Paracoccus denitrificans*. (A) The promoter of the *bioR*1 (*Pden_1431*) from *P. denitrificans*. (B) The promoter of the *bioYB* (*Pden_1432*) operon from *P. denitrificans*. (C) The promoter of the *bioR*2 (*Pden_2922*) from *P. denitrificans*. (D) The promoter of the *bioBFDAGC* (*Pden_2916*) operon from *P. denitrificans*. The predicted BioR site is given in cyan and underlined letter, and the possible ribosome binding site (RBS) is shown in purple and underlined type. The anticipated −10 and −35 regions are underlined in yellow. S, denotes transcription initiation site; M, denotes translation start site. **Figure S2.** Binding of *Paracoccus denitrificans* BioR1 (Pden_1433) to cognate promoters. (A) Binding of the *P. denitrificans* BioR1 (Pden_1431) the *Agrobacterium tumefaciens bioBFDAZ* promoter. (B) Binding of the *P. denitrificans* BioR1 (Pden_1431) its own promoter. (C) Interplay between the *P. denitrificans* BioR1 (Pden_1431) protein with the promoter of the *P. denitrificans bioBFDAGC* operon. (D) The *P. denitrificans* BioR1 (Pden_1431) protein interact with the *bioYB* operon. The BioR1 protein seemed unusual in that it very easily precipitates during the process of prepration in vitro, thus the crude extract of *Escherichia coli* overexpressing the Pden_1431 protein is used in the EMSA assays.
