# Supplementary material for: Paracoccus denitrificans possesses two BioR homologs having a role in regulation of biotin metabolism
Source: Microbiologyopen. 2015 Jun 2;4(4):644–59. doi: 10.1002/mbo3.270 (PMC4554459; doi:10.1002/mbo3.270)
Supplement: Supplementary file 1 [file mbo30004-0644-sd1.docx]

**Supplemental figures**

**
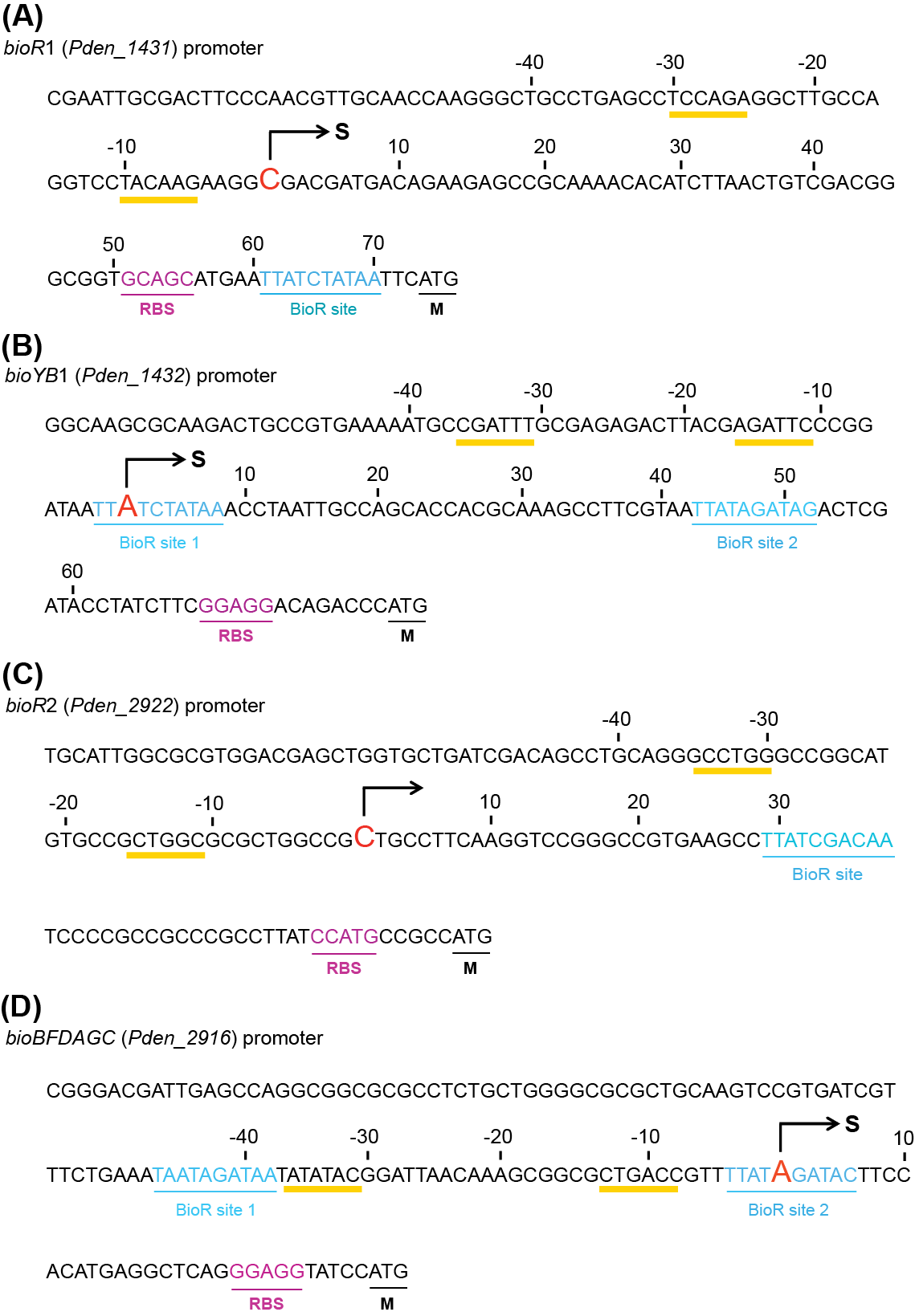
**

**Fig.S1** Molecular dissection for promoters of the *bio* operons from *Paracoccus denitrificans*

1. The promoter of the *bioR*1 (*Pden_1431*) from *Paracoccus denitrificans*
2. The promoter of the *bioYB* (*Pden_1432*) operon from *Paracoccus denitrificans*
3. The promoter of the *bioR*2 (*Pden_2922*) from *Paracoccus denitrificans*
4. The promoter of the *bioBFDAGC* (*Pden_2916*) operon from *Paracoccus denitrificans*

The predicted BioR site is given in cyan and underlined letter, and the possible ribosome binding site (RBS) is shown in purple and underlined type. The anticipated -10 and -35 regions are underlined in yellow. Abbreviations: S denotes transcription initiation site, and M denotes translation start site.


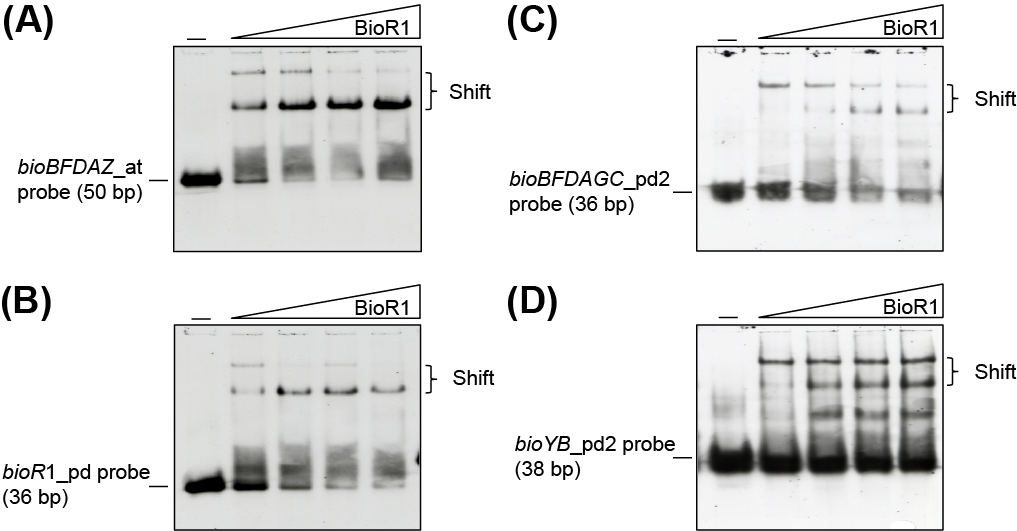


**Fig.S2** Binding of *P. denitrificans* BioR1 (Pden_1433) to cognate promoters

**A.** Binding of the *P. denitrificans* BioR1 (Pden_1431) the *A. tumefaciens* *bioBFDAZ* promoter

**B.** Binding of the *P. denitrificans* BioR1 (Pden_1431) its own promoter

**C.** Interplay between the *P. denitrificans* BioR1 (Pden_1431) protein with the promoter of the *P. denitrificans* *bioBFDAGC* operon

**E.** The *P. denitrificans* BioR1 (Pden_1431) protein interact with the *bioYB* operon

Note: the BioR1 protein seemed unusual in that it very easily precipitates during the process of prepration *in vitro*, thus the crude extract of *E. coli* overexpressing the Pden_1431 protein is used in the EMSA assays.
